# Engineering Yeast Extracellular Vesicle Biogenesis Through Rewiring Membrane Trafficking Pathways

**DOI:** 10.1111/1751-7915.70338

**Published:** 2026-03-27

**Authors:** Yueyan Li, XiaoRan Ma, Lichao Zhang, Ning Cao, Zhibo Li, Ruixin Khoo, Mei Wang, Changyan Li, Deping Hua, Xintian Zheng, Jinhai Huang, Lilin Zhang

**Affiliations:** ^1^ School of Life Sciences Tianjin University Tianjin China; ^2^ College of Life Sciences Longyan University Longyan China

**Keywords:** chicken interferon‐λ, engineering yeast, extracellular vesicles, *Saccharomyces cerevisiae*, SNARE components

## Abstract

Extracellular vesicles (EVs) are emerging as versatile therapeutic platforms, yet the mechanisms governing their biogenesis in yeast remain incompletely understood. *Saccharomyces cerevisiae* , a well‐characterised and safe microbial chassis, naturally secretes abundant EVs and provides an attractive system for mechanistic dissection and engineering. Here, we establish *S. cerevisiae* as a tractable model for elucidating EV cargo loading. By combining multicopy expression of chicken interferon‐λ (ChiIFN‐λ) with cell wall perturbation, we achieved a tenfold increase in EV yield and efficient incorporation of ChiIFN‐λ into EVs. Quantitative proteomics identified 1555 EV‐associated proteins, including 501 predicted transmembrane proteins derived from multiple organelles. ChiIFN‐λ overexpression and cell wall stress selectively reduced the abundance of key vesicle trafficking regulators, including SNARE, ESCRT and Rab proteins, indicating reprogramming of intracellular membrane trafficking pathways. Functional analyses further demonstrated that the SNARE proteins Sso2 and Nyv1 are enriched in the EV membrane and modulate EV size distribution and subpopulation composition. Together, these results reveal conserved protein‐sorting machinery underlying yeast‐derived extracellular vesicles (YDEVs) biogenesis and establish *S. cerevisiae* as a powerful platform for engineered EV production.

## Graphical abstract

Engineered *Saccharomyces cerevisiae* reveals how cell wall remodelling and membrane trafficking coordinate extracellular vesicle biogenesis. Interferon overexpression and *chs3* deletion enhance EV secretion and reshape SNARE‐, Rab‐ and ESCRT‐associated pathways, establishing yeast as a versatile platform for EV engineering and therapeutic cargo loading.

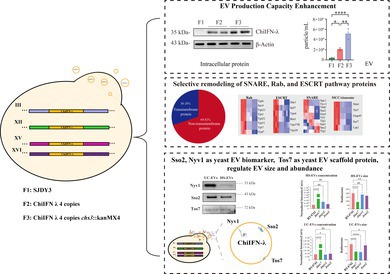

## 1 Introduction

EVs are membrane‐enclosed nanoparticles secreted by nearly all cell types, and serving as key mediators of intercellular communication. Based on their biogenesis, size and molecular composition, EVs are mainly classified as exosomes, microvesicles and apoptotic vesicles. Beyond these canonical categories, emerging subtypes such as migrasomes (Ma et al. 2015), supermeres (Zhang et al. 2021), mitovesicles (D'Acunzo et al. 2021) and midbody remnants (Peterman et al. 2019), have expanded the recognised heterogeneity of EVs (D'Angelo et al. 2025), highlighting their diverse cellular origins and functional complexity. To simplify the inherent complexity of EV classification across various organs and cell types, single‐celled eukaryotes are increasingly being considered as model systems. *S. cerevisiae* , in particular, has been widely used to study vesicle traffic and secretory system (Novick et al. 1980; Deshaies and Schekman 1987). Recent studies have reported that yeast, including both pathogens and non‐pathogens species from genera such as *Candida* spp. (Honorato et al. 2024; Kulig, Rapala‐Kozik, and Karkowska‐Kuleta 2024; Kulig, Wronowska, et al. 2024), *Cryptococcus* spp. (de Castro et al. 2023; Xiao et al. 2025; Castelli et al. 2025) and *Saccharomyces* spp. (Oliveira et al. 2010; Logan et al. 2024), can secrete EVs. Unlike EVs produced by other eukaryotic cells, fungal EVs have not been classified into distinct subpopulations (Brandt et al. 2024).

Concurrently, the identity of cargo proteins in YDEVs, particularly the biomarker proteins, remains poorly defined. EV cargo typically includes proteins, lipids, nucleic acids and other biomolecules, with composition varying significantly depending on the cell source (Welsh et al. 2024). These cargos reflect physiological and pathological states of their parent cells, offering invaluable insights into cellular behaviour, extracellular signalling and phenotypic changes. In mammalian‐derived EVs, the tetraspanins CD9, CD63 and CD81, along with ESCRT (endosomal sorting complexes required for transport) components such as Alix, TSG101 and syntenin, are well‐established biomarkers (Welsh et al. 2024; Jeppesen et al. 2019; Zhao et al. 2019). However, homologous proteins are absent from YDEVs. Although the ESCRT machinery is evolutionarily conserved across eukaryotes, and much of our current understanding coming from yeast models (Hurley et al. 2025), its role in YDEV formation remains poorly understood.

As a unicellular eukaryote, *S. cerevisiae* offers a genetically tractable and evolutionarily conserved system for dissecting EV biogenesis, free from the tissue‐specific variability observed in mammalian models. For thousands of years, yeast has been domesticated and utilised as a cell factory for the production of beer, wine and other food‐grade products, achieving high titre, rate and yield (TRYs) (Nielsen 2019). Further, extensive efforts have been made to enhance heterologous protein expression in yeasts at multiple levels, including transcriptional and translational control, secretion pathway engineering, optimisation of post‐translational modifications and metabolic reprogramming (Liu, Zhao, et al. 2025; Liu, Li, et al. 2025; Xie et al. 2007; Zhao et al. 2024). However, the incorporation of recombinant therapeutic proteins into YDEVs has not been demonstrated. Moreover, YDEVs are enriched in cell wall integrity (CWI) proteins and are abundant in mannans and glucans, which have been shown to exert potent anti‐inflammatory effects on the immune system (Brandt et al. 2024; Lee et al. 2021). These biological activations are crucial for enhancing immune responses against specific antigens, further underscoring the potential of engineered YDEVs as next generation drug delivery system.

Thus, herein we employed *S. cerevisiae* as an engineering platform to elucidate the molecular basis of EV biogenesis and cargo selection. Using recombinant strains expressing ChiIFN‐λ and cell wall‐deficient mutants, we isolated and characterised YDEVs. Proteomic profiling revealed multiple SNAREs (soluble N‐ethylmaleimide‐sensitive factor attachment protein receptor), ESCRT components and Rab GTPases (guanosine triphosphatase) enriched in these vesicles, suggesting conserved roles in vesicle trafficking. We further pinpointed Sso2 and Nyv1 as EV‐associated proteins potentially involved in cargo sorting or membrane fusion. These findings indicate that YDEVs utilise an evolutionarily conserved ESCRT‐Rab machinery for vesicle formation and establish *S. cerevisiae* as a tractable model for mechanistic EV research and bioengineering applications.

## 2 Materials and Methods

### 2.1 Plasmid Construction

To examine the expression and localisation of candidate EV proteins (Sso2, Nyv1 and Tos7), a series of tagged expression plasmids were constructed. The native promoter regions and open reading frames (ORFs) of target genes were amplified from the *S. cerevisiae* BY4741 genome and cloned into the Y42 vector at the EcoRI/SphI sites using seamless assembly (2 × MultiF Seamless Assembly Mix, Abclonal). This resulted in the construction of the following plasmids: Y42‐Sso2‐GFP, Y42‐Tos7‐GFP and Y42‐Nyv1‐GFP. The intron within the Nyv1 ORF was subsequently removed by reverse PCR using Nyv1‐specific intron‐deletion primers.

FLAG‐tagged constructs were generated by replacing the GFP coding sequence in the Y42 plasmid with a FLAG tag. The native promoter and ORF of Sso2 or Nhx1 were then inserted into the plasmid via EcoRI/XbaI, yielding the plasmids Y42‐Sso2‐FLAG and Y42‐Nhx1‐FLAG (deletion). HA‐tagged constructs, pHAC181‐Tos7‐HA and pHAC181‐Nyv1‐HA, were generated by cloning the promoter‐ORF fragments into the pHAC181 vector at EcoRI/SphI sites.

For *TEF2*‐Spo20PABD‐Y42, a 529‐bp *TEF2* promoter fragment was amplified from the JDY52 genome and inserted into the Y42 vector to create *TEF2*‐Y42. The Spo20 phosphatidic acid‐binding domain (Spo20PABD), containing an N‐terminal nuclear export signal, was generated by two‐step PCR. The GFP stop codon was removed by reverse PCR before performing a multiframe seamless assembly into the *TEF2*‐Y42 plasmid.

To generate the Y41‐based GFP fusion expression plasmid for Sso2 at the N‐terminal, the GFP stop codon was deleted from the Y41 backbone using primers anti‐F and anti‐R. An *Xho*I site was then inserted using primers to create Y41‐GFP‐XhoI. The Sso2 ORF was amplified from the Y42‐Sso2‐GFP vector using primers GFP‐Sso2‐Fo and GFP‐Sso2‐Ro and ligated with the linearised Y41‐GFP‐XhoI vector via homologous recombination, resulting in the plasmid Y41‐GFP‐Sso2‐ORF. Simultaneously, the Sso2 promoter was amplified from the Y42‐Sso2‐GFP vector using primers GFP‐Sso2‐Fp and GFP‐Sso2‐Rp. This promoter fragment was recombined with the linearised vector and transformed into *Escherichia coli* ( *E. coli* ) DH5α cells to yield plasmid Y41‐GFP‐Sso2.

Standard yeast strain culture and manipulation methods were used unless otherwise specified. The primers and plasmids utilised in this study are listed in Tables S2 and S3.

### 2.2 Engineering Yeast Construction

To investigate the incorporation of heterologous proteins into YDEVs, *S. cerevisiae* strains were engineered to express ChiIFN‐λ under the control of the constitutive *TDH3* promoter. Based on the multiplex CRISPR platform described previously (Baek et al. 2021), three intergenic loci on chromosomes III, XII and XV were selected as integration sites. A 23 bp synthetic DNA sequence containing a 20 bp guide RNA (gRNA) target (5′‐AAAGCGTCGCGCAATCGAGG‐3′) and a protospacer adjacent motif (PAM) was introduced into these loci in strain JDY52‐URR1‐His‐URR2 using CRISPR‐Cas9, yielding host strain SJDY3 (F1) with three gRNA‐binding sites.

Simultaneous integration of three ChiIFN‐λ expression cassettes into these sites was achieved under pCut plasmid editing via homologous recombination, and an additional copy was inserted between the *SUR1* and *PDR12* genes on chromosome XVI using the YeastFab rapid assembly method (Guo et al. 2015), generating the multicopy strain (F2). All integrations were confirmed by PCR genotyping with 100% accuracy.

To further assess the effects of cell wall remodelling on EV secretion, Chs3 encoding chitin synthase III, responsible for ~90% of cellular chitin synthesis (Sánchez and Roncero 2022), was disrupted by insertion of a *kan*MX4 cassette conferring G418 resistance. The resulting ChiIFN‐λ four‐copy *chs3Δ* mutant (F3) exhibited cell viability comparable to the wild type (data not shown). A complete list of plasmids and strains, including genotype and primer sequences, is provided in Tables S1–, S3.

To determine the co‐localisation of candidate proteins and specific organelle‐related proteins in YDEVs, a Vph1‐GFP‐tagged strain with endogenous recombinant was kindly provided by Professor Yongheng Liang (Chen and Liang 2023), in combination with the transformation of Nhx1‐Flag, Sso2‐Flag, Nyv1‐HA and Tos7‐HA plasmids in different transformants. Then the co‐transformed protein expression was confirmed by western blotting (WB) and performed density‐gradient ultracentrifugation as follows.

### 2.3 Yeast Growth and Microscopy Observation

Yeast strains were grown on yeast extract peptone dextrose (YPD) medium (1% yeast extract, 2% Bacto peptone, 2% glucose, w/o 2% agar) at 30°C for 2 days. If the strains were deletion mutants with a KanMX4 cassette, with an additional 100 μg/mL G418 for the plate.

Yeast transformants expressing GFP‐tagged proteins with URE3 selection marker were cultured on synthetic‐defined media lacking uracil (SD‐ura) medium at 30°C with shaking to mid‐logarithmic phase. Cells were harvested by centrifugation at 3000 × *g* for 5 min, washed twice with PBS and resuspended in PBS buffer for imaging. For vacuolar staining, FM4‐64 dye was added to a final concentration of 0.8 μM, and cells were incubated at 26°C for 2 h, followed by washing three times with PBS. Fluorescence images were acquired using a confocal laser scanning microscope (Leica STELLARIS 5, Germany) using Leica LAS X software.

### 2.4 Isolation and Purification of YDEVs

EVs were isolated from yeast culture supernatants using TFF (Tangential flow filtration) with differential ultracentrifugation or density‐gradient ultracentrifugation as described previously (Brennan et al. 2020). Yeast strains were first streaked from YPD or SD‐ura agar plates and incubated at 30°C for 3 days. Single colonies were used to inoculate 20 mL lipid media and cultured overnight at 30°C with shaking. The seed culture (50% v/v) was subsequently inoculated into 1 L fresh lipid medium in 2 L flasks and cultivated for 72 h. Then cells and large debris were removed by sequential centrifugation at 4000 *g* for 10 min at 4°C in Thermo Sorvall LYNX 6000 using T29 rotor. The resulting supernatant was filtered through a 0.45 μm Steritop vacuum filter (Millipore) to eliminate residual debris and concentrated using tangential flow filtration (TFF, 300 kDa molecular weight cutoff, Teomax, China). The medium was exchanged with phosphate‐buffered saline (PBS, pH 7.4). Then the concentrated filtrate was centrifuged at 20,000 × *g* for 1 h at 4°C using Thermo Sorvall in Fierlite F9 angle rotor to obtain high‐speed centrifugation EV fraction (HS‐EVs). The resulting supernatants were ultracentrifuged at 100,000 × g for 2 h at 4°C in Beckman Coulter Optima XPN‐100 ultracentrifuge, using Beckman Coulter Type79Ti rotor. The resulting pellet was washed once with cold PBS and resuspended in PBS to yield ultracentrifugation‐purified EVs fraction (UC‐EVs). Alternatively, the UC‐EVs pellets were further separated via sucrose density‐gradient ultracentrifugation. Briefly, the concentrated UC‐EVs were subjected to the top of a discontinuous density gradient consisting of 20% (w/v) to 60% (w/v) sucrose (2 mL volume for each angle) in particle‐free PBS solution. They were centrifuged at 100,000 × *g* for 14 h at 4°C using Beckman Coulter SW41ti rotor (stopping without break). Fractions (~500 μL) were collected from the top of the tube. 200 μL of each fraction was measured using an analytical balance with a readability of 0.1 mg using Sartorius BSA224S to determine the fraction density, and samples were stored at −80°C for storage.

### 2.5 Extracellular Vesicles Analysis

The EV size distribution and concentration were determined by Nano‐Flow cytometry (NanoFCM), dynamic light scattering (DLS) and nanoparticle tracking analysis (NTA) based on different situations. The particle size and number of YDEVs for mass proteomic analysis were characterised using NanoFCM instruments according to the manufacturer's instructions (NanoFCM Inc., Xiamen, China) (Lu et al. 2024). A silica nanosphere cocktail (cat. S16M‐Exo, NanoFCM Inc.) containing a mixture of 68, 91, 113 and 155 nm standard beads was used to adjust the instrument for particle size measurement.

For complementary size analysis, EV solution was filtered through 0.22 μm membranes, diluted in particle‐free PBS and tested by DLS (DynaPro NanoStar, US) and NTA (Malvern Panalytical NanoSight, NS300, Great Malvern, Worcestershire, England).

To determine the morphology, YDEVs samples were mounted on formvar–carbon‐coated copper grids (Beijing XXBR Technology, China) and negatively stained with 4% uranyl acetate in 7.5 mM oxalic acid (pH 7.0). EVs were observed and photographed under a transmission electron microscope (TEM) (Hitachi High‐Technologies Corporation, Tokyo, Japan). And the protein concentration of EV was determined using a bicinchoninic acid (BCA) kit (Solarbio) according to the manufacturer's instructions, without the use of detergents.

### 2.6 Proteinase K Treatment

To assess the topological distribution of proteins within EVs, protease protection assays were performed under native and permeabilised conditions. EV samples were thawed on ice and divided into three treatment groups: (1) untreated controls, (2) treated with 100 μg/mL proteinase K at 37°C for 60 min, and (3) pre‐treated with 1.5% Triton X‐100 on ice for 30 min prior to proteinase K digestion. Following incubation, protease activity was terminated by heating at 95°C for 5 min in 5 × SDS sample buffer. Treated samples were analysed by WB to evaluate the accessibility of EV‐associated proteins.

### 2.7 Antigen Preparation and Immunisation

Polyclonal antisera were generated to detect specific EV‐associated proteins. All animal experiments were reviewed and approved by the animal care and use committee of the institute of radiation medicine, Chinese Academy of Medical Sciences & Peking Union Medical College [approval number: IRM/2‐IACUC‐2409‐086, Tianjin, China]. Specific pathogen‐free (SPF) grade six‐week‐old female BALB/c mice were purchased from SPF Biotechnology Co. Ltd. [Beijing, Certificate Number: SCXK (Jing) 2019–0010] and housed in the SPF‐grade animal facility of the institute of radiation medicine, CAMS & PUMC, with a 12 h light/dark cycle, controlled temperature (22°C ± 2°C) and humidity (50% ± 10%). The mice were immunised subcutaneously with 50 μg of purified recombinant target protein emulsified in an equal volume of complete Freund's adjuvant (1:1, v/v). The emulsion was administered at three dorsal sites, followed by booster injections at 7 days intervals using incomplete Freund's adjuvant. Four days after the final immunisation, blood samples were collected via orbital sinus puncture. Serum was separated by centrifugation at 3000–4000 rpm for 20 min at 4°C and stored at −80°C until use.

### 2.8 Western Blotting

Extracellular vesicle suspensions were mixed with 5 × SDS sample buffer and heated at 100°C for 5 min. Yeast cell lysates were prepared by mechanical disruption with glass beads in lysis buffer (50 mM Tris–HCl, pH 8.0, 2 mM EDTA, 1 × PMSF), followed by centrifugation at 12,000 × *g* for 10 min at 4°C. Equal protein quantities were separated by SDS‐PAGE and transferred to polyvinylidene difluoride (PVDF) membranes (Millipore). Membranes were blocked in 5% non‐fat milk in TBST for 1 h at room temperature, incubated overnight at 4°C with primary antibodies, and then with HRP‐conjugated secondary antibodies for 1–2 h. Protein bands were detected using enhanced chemiluminescence (ECL) reagent (Super ECL, Yeasen, Cat. 36208ES76) and visualised using Alliance Q9 (Uvitec, UK) with a UVITEC system. Densitometric quantification was performed with ImageJ software.

### 2.9 Proteomic Analysis by LC–MS / MS

EV proteomes were analysed by Tingo Exosomes Technology Co. Ltd. in China (Yuan et al. 2024). Briefly, Liquid chromatography with tandem mass spectrometry (LC–MS/MS) was performed using Easy NLC 1200‐Q Exactive Orbitrap mass spectrometers (ThermoFisher). The nano‐HPLC system was equipped with an Acclaim PepMap nano‐trap column (C18, 100 Å, 75 μm × 2 cm) and an Acclaim Pepmap RSLC analytical column (C18, 100 Å, 75 μm × 25 cm). For this study, all spectra were collected in the positive mode using full‐scan MS spectra scanning in the FT mode from *m*/*z* 300 to 1650 at resolutions of 70,000. For tandem mass spectrometry (MS/MS) on the Q exactive (QE), the 15 most intense peptide ions with charge states ≥ 2 were isolated with an isolation window of 1.6 *m*/*z* and fragmented via higher‐energy collisional dissociation (HCD) with a normalised collision energy of 28. A dynamic exclusion of 30 s was applied.

The raw files were searched using Proteome Discover (version 2.1, Thermo Fisher, Germany) with Sequest as the search engine. The fragment and peptide mass tolerances were set at 20 mDa and 10 ppm, respectively, allowing for a maximum of 2 missed cleavage sites. The false discovery rates of proteins and peptides were 1%. Furthermore, differentially expressed proteins (DEPs) were analysed to identify the most significantly enriched signal transduction pathways using DAVID Bioinformatics Resource 2021 (https://david.abcc.ncifcrf.gov/).

### 2.10 Functional Enrichment Analysis

GO (Gene ontology) and KEGG (Kyoto encyclopedia of genes and genomes) pathway enrichment analyses were performed using the DAVID (Database for annotation, visualisation and integrated discovery) database (https://david.ncifcrf.gov/summary.jsp) with the *S. cerevisiae* S288C background. GO terms were classified into biological process (BP), molecular function (MF) and cellular component (CC) categories. The gene expression levels were analysed by the R‐based DESeq2 package. Significant up‐ and downregulated genes were identified using the false discovery rate (FDR), *p*‐value (*p* < 0.05) and log2 fold‐change (|log2foldchange| > 2).

### 2.11 Statistical Analysis

All experiments were performed in at least three independent biological replicates. Data are presented as mean ± SD. Statistical significance was determined by one‐way analysis of variance (ANOVA) using GraphPad Prism 5.0. A *p*‐value < 0.05 was considered statistically significant.

## 3 Results

### 3.1 Engineering *S. cerevisiae* Enhances EV Production and Alters Protein Cargo Composition

**FIGURE 1:**
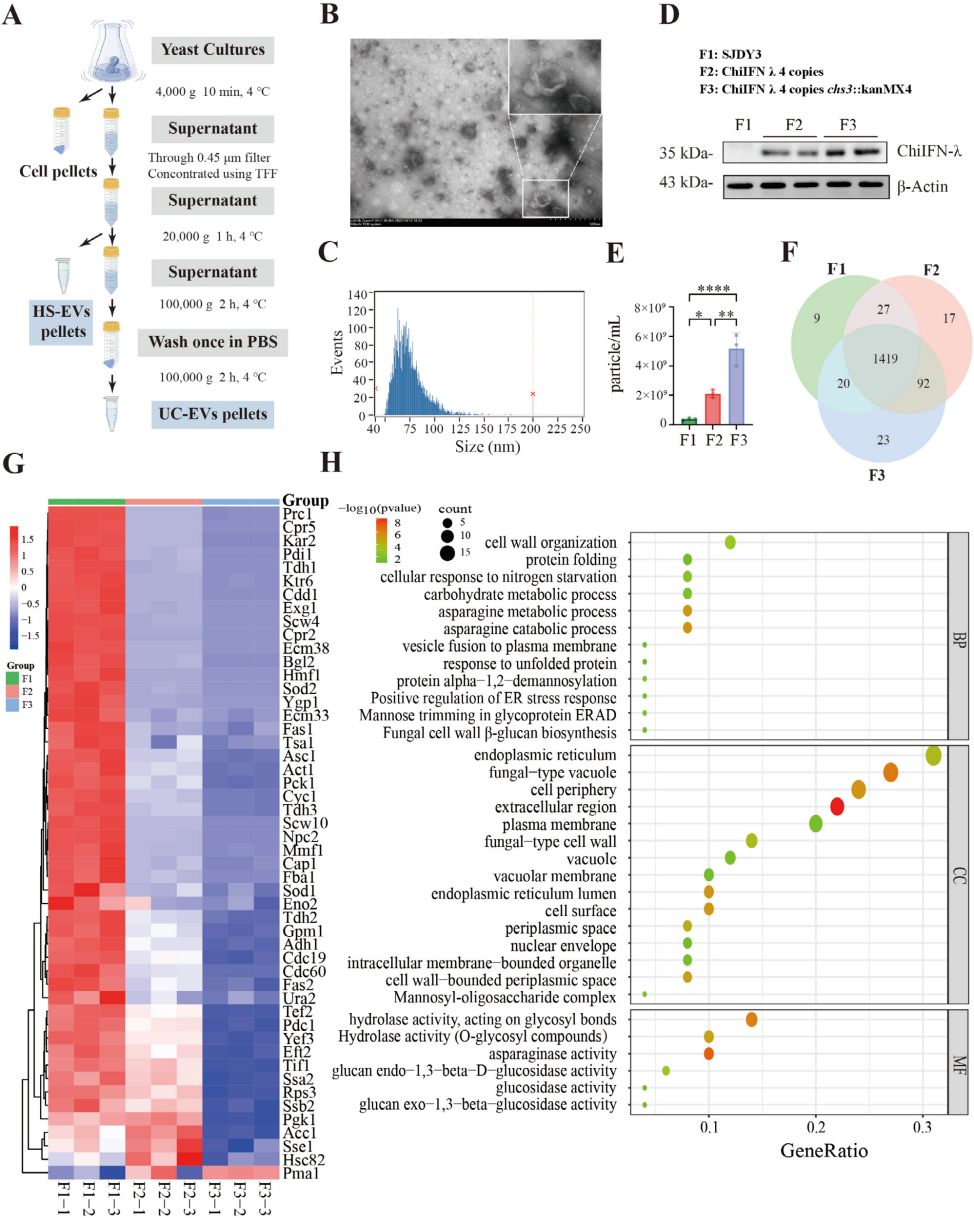
Engineering *S. cerevisiae* enhances EV production and reshapes protein cargo composition (A) Schematic workflow for the isolation of yeast‐derived EVs. (B) TEM images showing the morphology of isolated EVs (scale bar: 500 nm). (C) NanoFCM analysis of EV size distribution. (D) Immunoblot analysis of EVs obtained from each yeast strain. (E) Quantification of EV yield among the three engineered strains (*p < 0.05, **p < 0.01, ***p < 0.001, ****p < 0.0001). (F) Venn diagram of proteins repeatedly identified in the EV samples. Different colours represent three independent biological replicates within each group (F1‐EVs, F2‐EVs and F3‐EVs). (G) Heatmap showing the relative abundance of the top 50 most abundant proteins in F1‐EVs across the three strains. (H) GO enrichment analyses (cellular component, biological process and molecular function) of the top 50 most abundant proteins identified in F1‐EVs.

We engineered the wild *S. cerevisiae* strain SJDY3 (F1) by integrating the ChiIFN‐λ transcription unit into chromosomes III, XII, XV and XVI to generate a multicopy strain SJDY4 (F2). Subsequent deletion of *chs3*, which encodes chitin synthase III, yielded the cell wall‐deficient mutant SJDY5 (F3). EVs were purified from all strains (Figure 1A) and characterised using TEM, NanoFCM and mass spectrometry. UC‐EVs with diameters of 79 ± 15 nm were observed across all samples, consistent with exosome‐sized (Figure 1B,C; Figure S2B).

Western blotting confirmed the successful incorporation of exogenous ChiIFN‐λ into YDEVs (Figure 1D). NanoFCM analysis revealed that EV concentrations increased approximately 4‐ to 10‐ fold following genetic modifications (Figure 1E). The enhanced EVs production observed in the ChiIFN‐λ expressing and *chs3Δ* strains suggests that both intracellular stress responses and alterations in membrane architecture promote EV biogenesis in *S. cerevisiae* . Proteomic profiling identified 1555 proteins, of which 1419 were shared among all samples (Figure 1F). Among the shared components, the top 50 proteins derived from wild type yeast F1 strain exhibited significant decreased in abundance between ChiIFN‐λ expressing strain F2 and *chs3Δ* F2 groups (Figure 1G), indicating that ChiIFN‐λ expression and *chs3Δ* collectively enhance EV yield but altered endogenous protein‐loading patterns. Gene ontology enrichment analysis of these top elements indicated the top 50 cargos annotated to the endoplasmic reticulum, vacuole and plasma membrane (Figure 1H), consistent with the organelles reported to be involved in the sorting of mammalian exosomes in mammalian cells (Yuan et al. 2024). Together, these data indicate that genetic engineering and cell‐wall perturbation shift the EV proteome away from endogenous hydrolase‐rich protein toward heterologous proteins, implying remodelling of EV biogenesis and protein selection upon engineering EVs.

Collectively, these findings establish engineered *S. cerevisiae* as a tractable model for dissecting EV biogenesis and demonstrate its potential as a customisable platform for heterologous protein packaging and delivery.

### 3.2 Exogenous Protein ChiIFN‐λ Nonspecifically Loaded Into EVs

**FIGURE 2:**
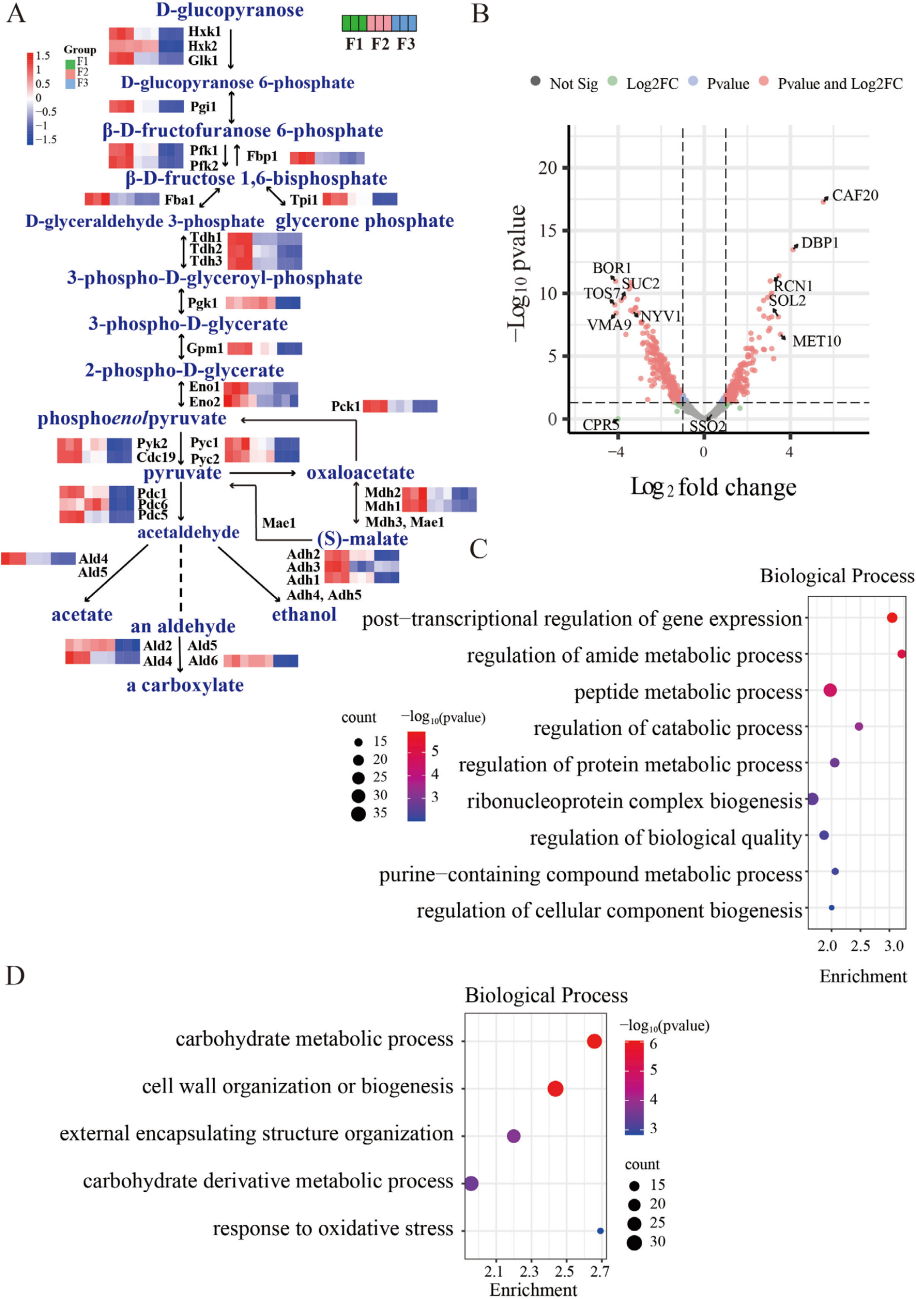
Overexpression of exogenous proteins significantly downregulated the glycolysis and gluconeogenesis related elements and altered the loading of functional cargoes in YDEVs. (A) Comparative analysis of the relative abundance of proteins associated with glycolysis and gluconeogenesis pathways (involving Tdh3) across EVs from different yeast strains. (B) Volcano plot showing quantitative differences in EV proteomes between F1 and F2 strains. The *x*‐axis (log_2_ fold change) represents the logarithmic transformation of relative expression changes between groups, where positive values indicate upregulation and negative values indicate downregulation in the experimental group. The *y*‐axis (−log_10_ *p*‐value) indicates statistical significance. Red dots denote proteins showing both significant fold change and statistical significance. (C) GO‐BP enrichment analysis of proteins upregulated in F2‐EVs compared with F1‐EVs. (D) GO‐BP enrichment analysis of proteins downregulated in F2‐EVs compared with F1‐EVs.

To investigate the effect of exogenous protein expression on the loading of YDEVs, the recombinant engineering *S. cerevisiae* strains carrying four copies of heterologous ChiIFN‐λ under the control of the endogenous *TDH3* promoter was constructed. Tdh3 encodes glyceraldehyde‐3‐phosphate dehydrogenase, a key enzyme in glycolysis and gluconeogenesis (Vande Zande et al. 2023). Comparative proteomic profiling revealed that most glycolysis‐ and gluconeogenesis‐related proteins were more abundant in wild‐type secreted EVs (F1) than in F2 and F3 engineered strains‐derived EVs (Figure 2A), suggesting that ChiIFN‐λ overexpression perturbs central carbon metabolism and further alters EV cargo composition.

Beyond glycolysis modifications, ChiIFN‐λ expression induced extensive proteomic remodelling. Specifically, 216 proteins associated with post‐transcriptional regulation, amide metabolism and peptide catabolism were upregulated (Figure 2B,C). These changes align with previous studies showing that heterologous proteins overexpression provokes translational stress and engages protein quality control mechanisms (Fujita et al. 2025; Hou et al. 2012a, 2012b; Wheeler et al. 2017; Groll et al. 1997; Maxwell et al. 2021). Additionally, Caf40, Not5 and other nine kinds of P‐body components were significantly enriched in F2 group (Figure S1A). P‐bodies are cytoplasmic ribonucleoprotein (RNP) granules involved in mRNA storage and turnover. Upon overexpression of exogenous proteins, the yeast translation system becomes highly occupied, thereby impairing the efficient translation of endogenous mRNAs. As a result, excess untranslated mRNA sequestered into P‐bodies, leading to increased abundance of P‐body associated proteins. Accordingly, elevated level of P‐body proteins serves as an indicator of intracellular mRNA accumulation arising from inefficient translation (Lavut and Raveh 2012; Wang et al. 2018). In addition, several proteasomal subunits, including Pre3, Pre6 and Pre8, exhibited enrichment patterns similar to those of P‐body components (Figure S1B). These finding indicate that heterologous protein expression may induce cellular side effects, such as translational arrest, RNA‐protein condensate formation and the rerouting of stress‐related material into EVs. In contrast, 234 downregulated proteins were enriched in carbohydrate metabolism, cell wall biogenesis, external structure organisation and oxidative stress (Figure 2B,D). Taken together with Figure 2A, the downregulation of central glycolysis enzymes reflected a cellular shift away from energy production toward stress adaptation, consistent with an ‘energy‐conservation’ response that reallocates resources to protein quality control and homeostasis (Wagner et al. 2007; Liu, Zhao, et al. 2025; Liu, Li, et al. 2025).

**FIGURE 3:**
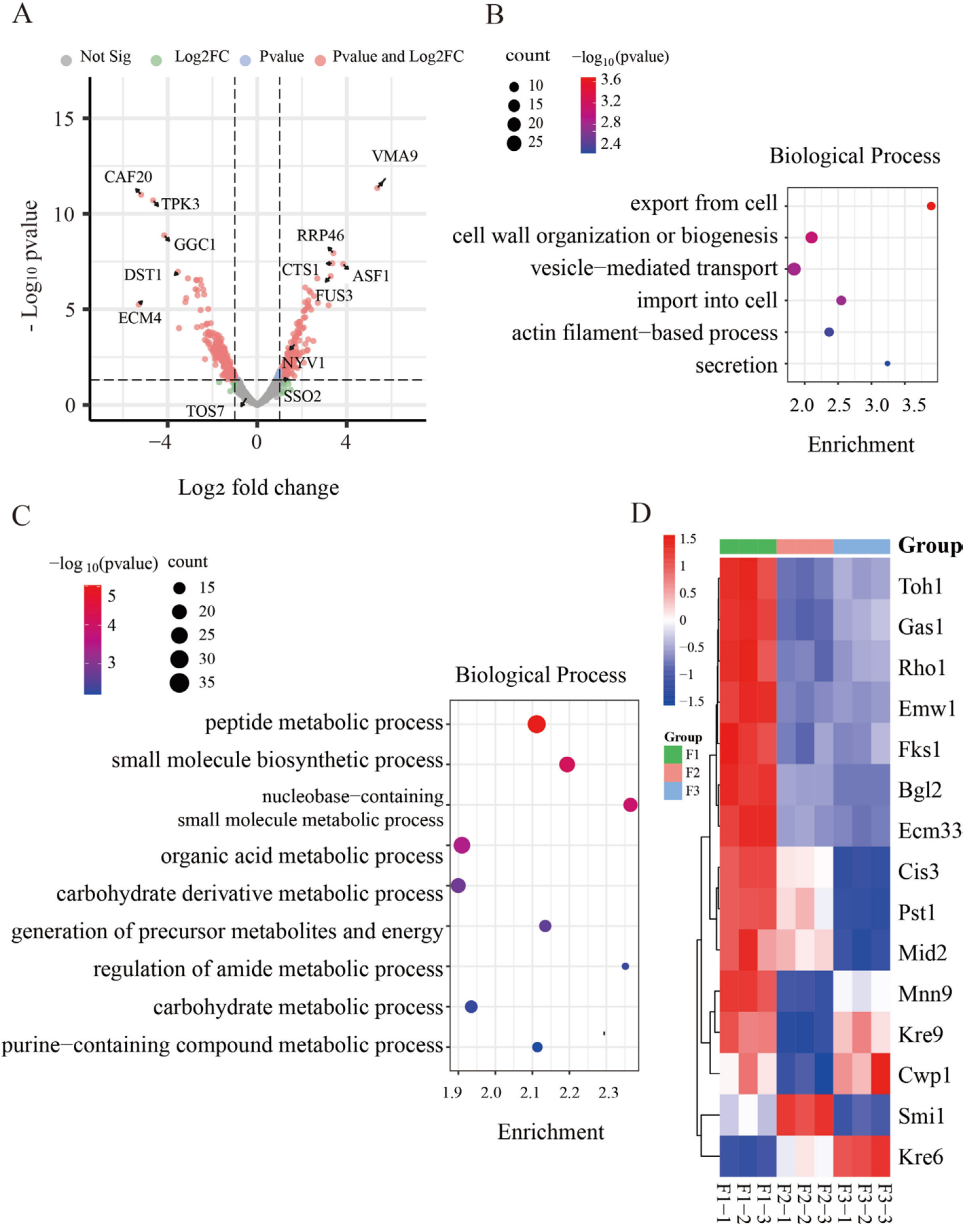
Knockout of *chs3* further remodelled cargo composition by altering cell‐wall synthesis. (A) Volcano plot showing quantitative differences in EV proteomes between F2 and F3 strains, with axes and colour coding as described in Figure 2B. (B) GO‐BP enrichment analysis of proteins upregulated in F3‐EVs compared with F2‐EVs. (C) GO‐BP enrichment analysis of proteins downregulated in F3‐EVs compared with F2‐EVs. (D) Heatmap illustrating abundance changes of proteins involved in the CWI signalling pathway and cell wall assembly among different strains.

To further explore the impact of cell‐wall integrity on YDEV secretion and ChiIFN‐λ loading, Chs3, encoding chitin synthase III responsible for ~90% of cellular chitin synthesis (Sánchez and Roncero 2022) was deleted in the ChiIFN‐λ multicopy strain to generate the F3 mutant. Previous studies have shown that *chs3* disruption increases EVs release in *S. cerevisiae* (Zhao et al. 2019), whereas in *C. neoformans* it reduces capsule size and EV output (Rodrigues et al. 2018). Consistently, knockout of *chs3* in our system enhanced the yield of YDEVs (Figure 1E) and further remodelled cargo composition by altering cell‐wall synthesis and actin‐dependent vesicle transport. Upregulated proteins in F3‐EVs were enriched for vesicle‐mediated transport, secretion and cell‐wall organisation (Figure 3A,B), while downregulated proteins were primarily involved in carbohydrate metabolism and small‐molecule biosynthesis (Figure 3A,C), reflecting a metabolic shift toward vesicle formation and membrane remodelling. Given that Chs3 is a direct transcriptional target of Rlm1 in the CWI signalling pathway (Jiang et al. 2018; Du et al. 2026), *chs3* loss likely perturbs CWI‐mediated stress responses. Interestingly, despite the overall downregulation of CWI pathway components (Figure 3D), several downstream effectors, such as Kre6, Kre9 and cell surface sensor Cwp1, were upregulated after *chs3Δ* mutant, suggesting compensatory activation of β‐1,6‐glucan synthesis encoding by Kre6/9 to maintain wall integrity is necessary. Collectively, these findings indicate that exogenous ChiIFN‐λ expression and *chs3* deletion synergistically remodel cellular metabolism and YDEVs biogenesis through coordinated metabolic reprogramming, stress adaptation and compensatory cell‐wall responses. To further elucidate how these perturbations reshape YDEVs formation, we analysed the molecular machinery involved in vesicle trafficking and membrane organisation, focusing on SNAREs, Rab GTPases and ESCRT‐associated proteins.

### 3.3 Mass Spectrometry Reveals a Conserved Vesicle‐Trafficking Components in YDEVs

It has been suggested that the extensive metabolic reprogramming and cell‐wall remodelling could influence the cargo loading of EVs. From this perspective, we sought to determine how these cellular perturbations influence EV formation and membrane composition. EVs are enclosed by lipid bilayers that mirror the characteristics of the originating cell's plasma membrane or organellar membrane. In fungi such as *Cryptococcus* spp., EVs are further decorated by mannoprotein‐based fibrillar structures (Rizzo et al. 2021). While in mammalian systems, EVs exhibit substantial morphological heterogeneity, including spherical, tubular and double‐membrane vesicles derived from tumorigenic cells (T3M4), nontumorigenic cells (HEK293T) and human serum samples (Kapoor et al. 2024). This structural diversity strongly suggests the coexistence of multiple biogenetic routes and heterogeneous subcellular membrane origins (Neyroud et al. 2022; Malenica et al. 2021; Zabeo et al. 2017; Li et al. 2025).

**FIGURE 4:**
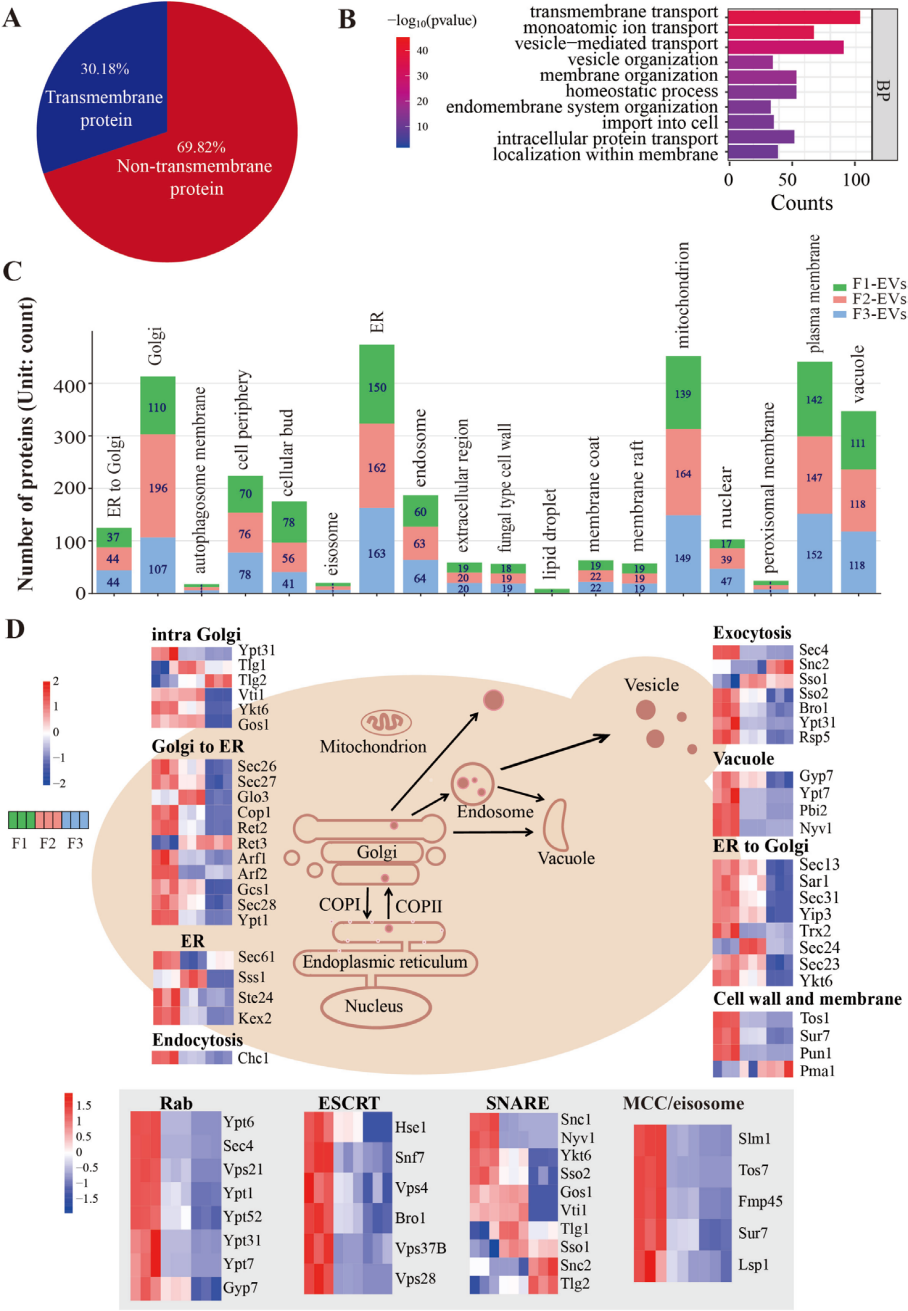
Proteomic analysis reveals conserved vesicle‐trafficking components in YDEVs. (A) Proportion of transmembrane proteins among all proteins identified in F1‐EVs. (B) GO‐BP enrichment analysis of transmembrane proteins. (C) Comparative subcellular localisation of transmembrane proteins in F1‐EVs, F2‐EVs and F3‐EVs. Bars indicate the number of proteins assigned to each subcellular compartment. (D) Heatmap showing relative abundance changes of vesicle trafficking regulators, including ESCRT components, SNARE proteins, Rab GTPases and plasma membrane compartment of Can (MCC)/eisosome‐associated proteins.

In this context, EV morphology provides structural evidence for differences in membrane source and assembly mechanism, while proteomic profiling enables molecular inference of these origins. Guided by this framework, we focused on the membrane‐associated proteome and subcellular localisation signatures of YDEVs. Using Uniport annotations and a membrane protein prediction online tool called SOSUI, 501 proteins containing at least one transmembrane domain were detected, representing 30.2% of all EV proteins (Figure 4A). These proteins were primarily assigned to the endoplasmic reticulum (ER), mitochondria, plasma membrane, Golgi apparatus and vacuole (Figure 4B,C). Notably, more than 110 membrane proteins were annotated as vacuole, suggesting a contribution of vacuolar membranes to the YDEV population. Recombinant protein markedly altered the membrane proteome landscape. Specifically, membrane protein localisation shifted toward Golgi, ER, mitochondrial and nuclear annotations in engineered strains (Figure 4C and Table S4). This redistribution coincided with known cellular responses to heterologous protein expression, which impose a substantial burden on protein biogenesis and trafficking pathway, subsequently triggering the unfolded protein response (UPR) activation, ER membrane expansion and enhanced vesicular transport capacity (Travers et al. 2000; Schuck et al. 2021; Hou et al. 2012a, 2012b; Bao et al. 2018). Increased secretory flux can compromise retrograde retrieval and quality control systems, thereby altering cargo selection within endosomal and multivesicular body (MVB) pathways. Under these conditions, ER‐and Golgi‐resident membrane proteins may be mis‐sorted or preferentially incorporated into intraluminal vesicles, reshaping the membrane protein composition of YDEVs. In parallel, elevated metabolic demand and proteostatic stress associated with recombinant protein overproduction have been reported to remodel organelle contact sites, including ER‐mitochondria interfaces, which may influence mitochondrial membrane dynamics and stress‐associated vesicular export (Bao et al. 2018; Castro et al. 2022; Ishiwata‐Kimata and Kimata 2023). The enrichment of proteins annotated as nuclear is likely attributable to expansion of the ER‐nuclear envelope continuum, given the structural continuity between these compartments, rather than direct nuclear membrane contribution. Correspondingly, proteins assigned to endosome and plasma membrane compartments showed a modest increase in YDEVs derived from engineered strains. These trends were particularly pronounced among the 50 most abundant membrane proteins (Figure S1C), and minimal overlap was observed among membrane protein sets from the three experimental strains (Figure S1D). Collectively, these data indicate that recombinant protein expression reprograms intracellular membrane trafficking networks and alters the relative contribution of membrane sources to EV biogenesis, highlighting the plasticity of vesicle formation pathways under synthetic engineering conditions (Mattanovich et al. 2004).

To investigate EV biogenesis, we analysed trafficking regulators detected in EV preparations, including ESCRT components, SNARE proteins, Rab GTPases and eisosome‐associated factors (Figure 4D). Although yeast encodes around 20 ESCRT‐related proteins (Brune et al. 2019; Remec Pavlin and Hurley 2020), prior work reported the absence of several ESCRT components in wild‐type YDEVs (Zhao et al. 2019), implying mechanistic divergence from canonical mammalian exosome formation. In our dataset, six ESCRT‐associated proteins were consistently identified: Vps37 and Vps28 (ESCRT‐I), Snf7 (ESCRT‐III), Hse1 (ESCRT‐0) and the ESCRT‐associated proteins Bro1 and Vps4. These proteins were relatively enriched in wild‐type EVs. Hse1, which functions in Golgi‐to‐endosome retrieval and sorting of ubiquitinated cargo (Morshed et al. 2020; Bilodeau et al. 2002), has previously been shown to influence EV protein composition (Zhao et al. 2019). In the F2 strain, interferon overexpression significantly altered the EV proteome. Specifically, the loading abundance of ESCRT, including both core and accessory components, was markedly decreased (Figure 4D). Although studies in mammalian systems indicate that accessory ESCRT proteins regulate exosome biogenesis (Marie et al. 2023), their abundance in EVs from the engineered strain decreased in parallel with the core ESCRT components in our dataset. Previous work has shown that amino acid starvation activated the TORC1 (TOR complex 1) signalling, which indirectly suppresses ESCRT‐dependent pathways and links vesicle trafficking to cellular metabolic status (Babst and Odorizzi 2013). Consistent with this framework, our results indicate reduced reliance on classical ESCRT‐dependent MVB sorting under interferon overexpression conditions. In the F3 strain, deletion of *chs3* further reduced ESCRT protein abundance. Loss of Chs3 compromises CWI and promotes EV release via direct plasma membrane budding rather than MVB‐mediated secretion, thereby further diminishing ESCRT involvement. Together, interferon overexpression and *chs3* deletion shift EV production from ESCRT‐dependent toward ESCRT‐independent pathways. The differential reduction among individual ESCRT components likely reflects their relative contributions to EV biogenesis.

The SNARE complex mediate fusion of MVBs with the plasma membrane during exosome secretion (Liu et al. 2023). In this study, 10 of the 24 canonical yeast SNAREs proteins were identified in YDEVs (Menaceur et al. 2023). They are Qa‐SNASE Sso1, Sso2 and Tlg2, Qb‐SNARE Gos1 and Vti1, Qc‐SNARE Tlg1 and R‐SNARE Ykt6, Nyv1, Snc1 and Snc2. Consistent with previous reports, Sso2 and Ykt6 have also been detected in EVs from *Candida albicans* (Dawson et al. 2020), supporting their conserved association with EV. Overexpression of ChiIFN‐λ resulted in a marked reduction in the abundance of Snc1, Nyv1, Ykt6, Sso2, Gos1 and Vti1 in YDEVs, indicating perturbation of both secretory and vacuolar fusion machinery. Notably, the detection of the vacuolar SNAREs Nyv1 within YDEVs supports the contribution of vacuole‐derived membranes to the secreted vesicle pool. Distinct regulatory patterns were observed in the *chs3Δ* background. Ykt6, Sso2, Gos1 and Vti1 were exclusively downregulated, whereas Snc2 and Tlg family proteins were selectively upregulated. Chs3 is a chitin synthase III, which is required for the majority of cell wall chitin synthesis. Its deletion leads to CWI stress and enhanced EV production (Shaw et al. 1991; Levin 2011; Grote et al. 2000). The R‐SNARE Ykt6 is a key regulator of exosome biogenesis and release, and its membrane association is essential for exosomal sorting. Previous studies in neurodegenerative disease models have shown that impaired membrane anchoring of Ykt6 reduces exosome secretion (Tsunemi et al. 2025). Given the high conservation of Ykt6 across eukaryotes, the decreased Ykt6 content observed in YDEVs likely reflects compromised membrane binding under the combined stresses of heterologous protein overexpression and cell wall damage. Another components, Sso proteins are yeast homologues of syntaxins and function in fusion of secretory vesicles with the plasma membrane. Although Sso1 is required for sporulation (Jäntti et al. 2002; Sugiyama and Tanaka 2019), Sso2 interacts with the stress sensor Wsc1 in the CWI signalling pathway (Santiago‐Cartagena et al. 2019). The increased loading of Sso1 in YDEVs derived from ChiIFN‐λ expression and *chs3Δ* strain may therefore be linked to enhanced secretory vesicles trafficking during bud formation, whereas the selective reduction of Sso2 in *chs3Δ* EVs is consistent with stress‐dependent feedback regulation through the CWI pathway. Gos1 and Vti1 participate primarily in intra‐Golgi (Tsui and Banfield 2000) and Golgi‐endosome/vacuolar trafficking routes (Fischer von Mollard and Stevens 1999). Their decreased abundance in *chs3Δ* derived YDEVs suggests reduced contribution of canonical endomembrane and retrograde trafficking pathways to EV biogenesis under cell wall stress. Activation of the CWI pathway may reprogram vesicle cargo toward components involved in cell wall repair (Jiang et al. 2018), resulting in the relative underrepresentation of Gos1 and Vti1 within the limited vesicular lumen. In addition, Vti1 mediates cyclic trafficking of Chs3 between the trans‐Golgi network, endosomes and the plasma membrane (Deng et al. 2009), disruption of this trafficking loop in the *chs3Δ* mutant likely further contributes to reduced Vti1 incorporation into EVs. Snc1 and Snc2 are highly abundant SNAREs that continuously cycle through secretory and endosomal systems. Snc1 functions in post‐endocytic recycling (Best et al. 2020), whereas Snc2 is preferentially enriched in budding regions (Sugiyama and Tanaka 2019). The selective enrichment of Snc2 in *chs3Δ* YDEVs is therefore consistent with compensatory increases in bud‐directed secretory trafficking triggered by CWI activation. Tlg proteins, which localise to endosome‐plasma membrane interfaces (Holthuis et al. 1998), were also enriched in *chs3Δ* EVs, supporting a shift toward enhanced exocytic and plasma membrane derived vesicle formation. Collectively, disruption of chitin synthesis and activation of compensatory CWI responses result in pathway‐specific remodelling of the SNARE network in YDEVs. These changes reflect trafficking reprogramming rather than global proteomic fluctuations, highlighting selective modulation of vesicle biogenesis routes under combined secretory and cell wall stress.

The Rab family of small GTPases controls all aspects of intracellular vesicle trafficking such as coordinators of vesicle biogenesis, motility, tethering and fusion (Menaceur et al. 2023; Heo et al. 2021). Eight out of the 11 yeast Rab proteins, including Ypt1, Ypt6, Sec4, Ypt31/52, Ypt7, Vps21 and Gyp7, were detected in EVs (Figure 4D and Table S4). Most Rab proteins showed reduced abundance following ChiIFN‐λ overexpression, with a further decrease observed in *chs3Δ* derived EVs. Together, these findings suggest that exogenous protein expression and cell‐wall disruption jointly remodel vesicle trafficking pathways and membrane composition, reshaping the molecular landscape of YDEVs.

In addition to ESCRT, SNARE and Rab machineries, proteomic analysis identified several components of the plasma membrane compartment of Can1 (MCC)/eisosome microdomains that were differentially represented in the YDEVs (Figure 4D). These membrane domains are critical for plasma membrane organisation, cell wall homeostasis and stress signalling (Olivera‐Couto et al. 2011; Berchtold et al. 2012; Sakata et al. 2022; Zhu et al. 2020). Notably, MCC/eisosome‐associated proteins were generally depleted in EVs from the *chs3Δ* strain, which displays compromised cell wall integrity and elevated EV production. This finding suggests that the loss of chs3 and the resulting cell wall stress reconfigures plasma membrane microdomains (Shaw et al. 1991; Levin 2011), thereby reducing the incorporation of eisosome‐associated proteins into EVs. Collectively, these data support a model in which *chs3Δ* redirects EV biogenesis away from canonical microdomain‐dependent pathways.

### 3.4 SNARE Components Sso2 and Nyv1 as Candidate Biomarker for Yeast‐Derived EVs

**FIGURE 5:**
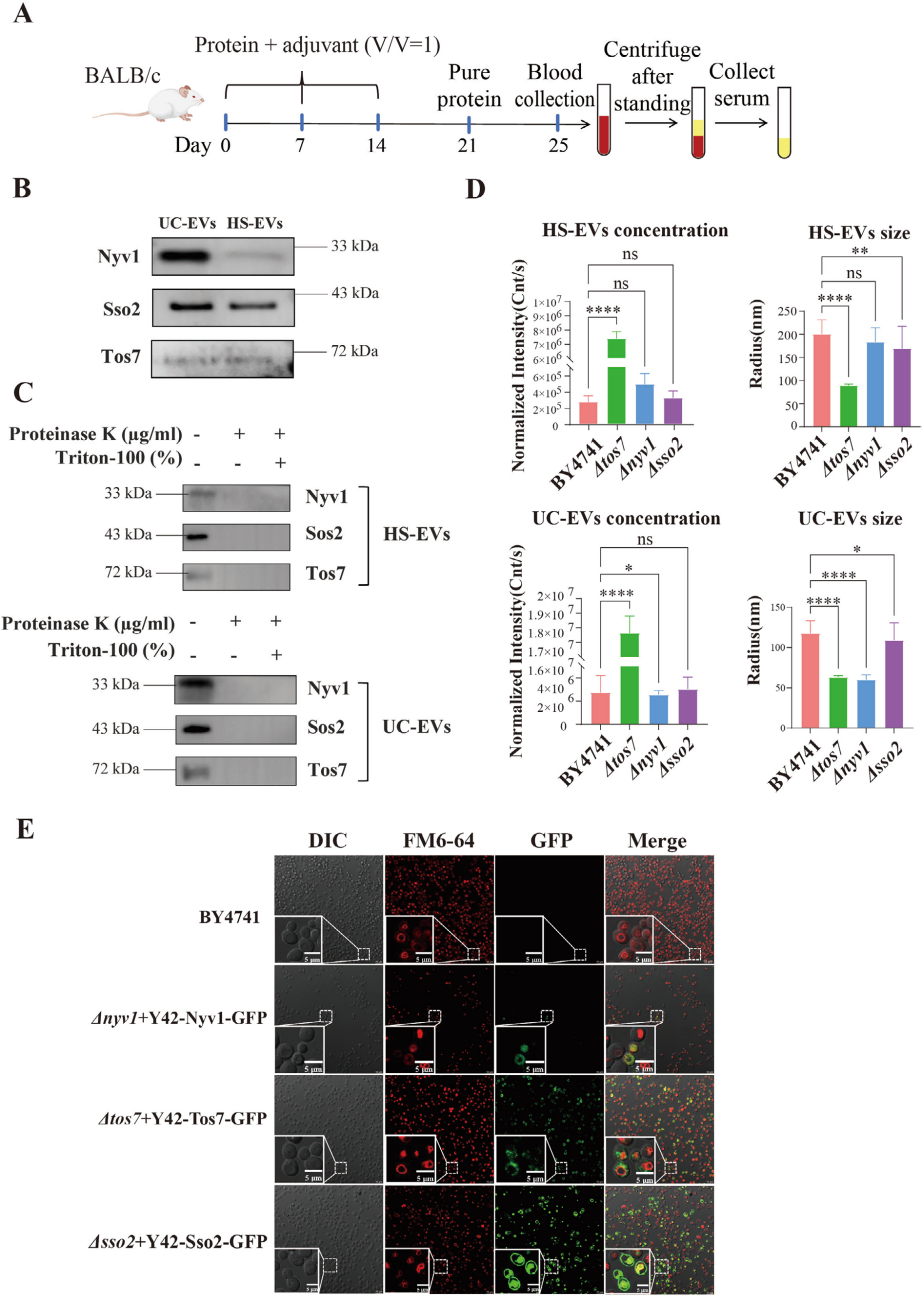
Functional interrogation of membrane proteins Sso2, Nyv1 and Tos7 in yeast EVs. (A) Schematic diagram illustrating the preparation process of specific polyclonal antibodies against the target proteins. (B) Detection of target proteins in EVs derived from wild‐type *S. cerevisiae* (BY4741) using the custom‐generated specific antibodies. (C) Determination of the localisation of target proteins within the EV lumen or on the EV surface. (D) Analysis of particle size distribution and concentration of HS‐EVs and UC‐EVs by DLS (*p < 0.05, **p < 0.01, ***p < 0.001, ****p < 0.0001). (E) Fluorescence microscopy images of wild‐type yeast and recombinant yeast strains expressing GFP‐fusion constructs, stained with FM4‐64 (red). Dashed boxes indicate regions shown at higher magnification (scale bar: 10 μm).

Based on the global redistribution of vesicle trafficking machinery observed above, we next focused on two representative SNARE proteins, Sso2 and Nyv1. Both proteins are core components of the vesicle fusion system and were consistently detected with high abundance in YDEV proteomes across conditions, making them suitable candidates for experimental validation and potential EV biomarkers. WB analysis using homemade mouse‐derived antibodies and commercially available anti‐Flag/HA tag antibodies confirmed that these two proteins were present in UC‐EVs fractions, consistent with our previous proteomics data (Figure 5A,B; Figure S2C). Next, we analysed the subcellular localisation of the two candidate biomarkers. Proteinase K treatment indicates that the candidate cargos are accessible on the EV surfaces, consistent with a membrane‐associated localisation (Figure 5C). Further, the proportion of cargo protein‐carrying positive EVs relative to the total EVs was tested using yeast transformants carrying Y42‐Sso2/Nyv1‐GFP plasmids by fluorescent mode NTA. The mean concentration of GFP EVs was measured as 1.2 × 10 E + 09 particles/mL (take up ~5%) in UC‐EVs and 1.08 × 10 E + 08 (~1%) in HS‐EVs of Sso2, while Nyv1 positive vesicles take up 1% in UC‐EVs and 10% in HS‐EVs. Collectively, these findings nominate Sso2 and Nyv1 as specific markers of yeast‐derived EVs and as candidate scaffold for EV engineering. In addition, MCC/eisosome component Tos7 was also identified in EVs compounds (Figure 5B and Figure S2C). As shown in Figure 5C, Tos7‐HA predominantly exposed on the surface of HS‐EVs fractions, and further depleted with proteinase K treatment.

Furthermore, we explored the impact of the deletion of these candidate proteins on YDEVs production. *tos7Δ* mutant produced a marked increase in both EV concentration accompanied by a reduction in mean vesicle diameter (Figure 5D). This phenotype is consistent with a role for Tos7 in eisosome organisation and cell‐wall architecture. In contrast, *nyv1Δ* yielded a modest but statistically significant reduction in UC‐EV concentration as well as a significantly decreased UC‐EV diameter, which indicated Nyv1 primarily influences UC‐EV biogenesis. Another SNARE component, *sso2Δ*, did not alter EV concentration but produced a pronounced decrease in vesicle diameter consistent with partial functional compensation by Sso1. Notably, the above data tested by DLS, derived diameters of vesicles were consistently larger than measured by NTA and NanoFCM, whereas the concentration obtained by DLS is generally lower than NTA and NanoFCM (Rodriguez‐Loya et al. 2023; Filipe et al. 2010; Zhang et al. 2025). Given the discrepancies among the DLS data, NTA data and the NanoFCM values, it is sufficient to carry out a horizontal comparison of EVs concentrations and size between mutant and wild type strains, and unnecessary to perform a longitudinal comparison across different measurement batches.

Subsequently, fluorescence microscopy further confirmed the subcellular distribution of these proteins, revealing that Nyv1 and Tos7 partly co‐localised with the vacuolar marker FM4‐64 (Figure 5E), consistent with the proteomic annotations. Surprisingly, Sso2‐GFP was intracellularly localised (Figure 5E), in accordance with endogenous Sso2 imagines via homemade mouse anti‐Sso2 and mouse anti‐FITC antibodies yielded a similar subcellular location (Figure S2A), but distinguished with GFP‐Sso2 in the plasma membrane (Figure S2A) as shown on LoQAtE (https://www.weizmann.ac.il/molgen/loqate/YMR183C) and Rima's study (Mendonsa and Engebrecht 2009), and the exact regulatory mechanism remains to be fully elucidated.

Given that the proteins localised in plasma membrane or vacuole, and their presence in EV fractions was verified by WB, we lastly sought to clarify their sorting pathways and origins. Homologous organelle markers from known mammalian exosome sorting pathways, including late endosome, vacuole and plasma membrane markers (van Niel et al. 2018), were selected as references to detect candidate protein co‐localisation in YDEVs through density gradient centrifugation. The results are shown in Figure S2D, the distribution of Sso2 and Nyv1 cargos was all distributed in fractions 12–19, corresponding density from 1.15 to 1.22 g/mL, in alignment with previous reports (Lu et al. 2024; Winters et al. 2020). Taken together, these data indicate that SNARE complex and eisosome‐associated proteins influence EV morphology and population heterogeneity, and identified Sso2 and Nyv1 as promising markers and handles for future yeast EV scaffold engineering.

## 4 Discussion

EVs are emerging as promising vehicles for transformative opportunities in personalised medicine, diagnostics and targeted therapies (Wang et al. 2025; Ripoll et al. 2026). EVs derived from mesenchymal stem cells (MSCs), pluripotent stem cells (iPSCs), serum and plasma have been actively researched for their immunomodulatory properties and trauma‐repairing potential in regenerative medicine (Li et al. 2024). However, the inherent heterogeneity of EVs presents a significant challenge to their clinical translation, despite increasing emphasis on the importance of single‐vesicle analysis techniques (Zhang et al. 2025). Furthermore, exosomes face critical limitations, particularly regarding yield optimisation and economic feasibility. As previously noted, utilising mass‐cultivable probiotics for EVs production offers a promising solution to these bottlenecks (Herrmann et al. 2021). In addition, this approach can significantly enhance the cost‐effectiveness of the process, from cell culture to isolation and improve scalability, thereby providing a solid foundation for the clinical application of probiotic exosome‐based delivery systems in precision medicine.

In the present study, we successfully detected the non‐specific incorporation of ChiIFN‐λ into YDEVs, through the engineered *S. cerevisiae* strains overexpressing secretory ChiIFN‐λ. This finding shed light on the potential for future YDEVs engineering applications. Current research on exosome modification predominantly focuses on two approaches: passive and active strategies (Lee et al. 2024). Passive approaches involve loading exogenous protein into exosome after secretion via techniques such as incubation, electroporation, sonication, extrusion, freeze–thaw cycling; inadequate efficiency still persists (Zeng et al. 2023). In contrast, active strategies integrate functional molecules directly into the exosome scaffolds through genetic engineering. Ideally, exosome engineering scaffolds should facilitate the efficient packaging of multiple protein copies without disrupting the exosome biogenesis pathway or its luminal content. However, research on probiotic‐derived exosomes remains in early stages, with several critical challenges yet to be addressed, including the identification of suitable biomarkers and scaffold proteins. Thus, the present study further investigated candidate tags for YDEVs. Given that these candidates contain transmembrane domains, they show significant potential as scaffold proteins for the targeted loading and delivery of various proteins.

Biomarkers are crucial for the purification, identification and genetic recombination of YDEVs. As widely recognised, the tetraspanins CD9, CD81 and CD63 are among the most widely used as EVs markers and regulate the sorting of cargo proteins into EVs (Zheng et al. 2023). However, homologous proteins are absent in yeast cells. According to the international society for extracellular vesicles (MISEV2023) guidelines, three key criteria must be met for a protein to qualify as an EV marker: (1) It must be strongly associated with exosome biogenesis and stably expressed. The protein should be involved in EV formation, and deletion mutants should impair sorting or secretion of YDEVs (van Niel et al. 2018; Zhang et al. 2024). In this study, the SNARE components Sso2 and Nyv1 were stably expressed in both HS‐EVs and UC‐EVs. Loss‐of‐function studies indicated that the endosome‐localised Qa‐SNARE Sso2 primarily affects vesicle diameter, while the R‐SNARE Nyv1 modulates the formation and release of UC‐EVs linked to vacuolar and plasma membrane trafficking. Furthermore, density gradient ultracentrifugation results confirmed that Sso2 distribution profiles closely match those of the late endosomal membrane protein Nhx1, supporting its involvement in the late endosome‐associated biogenesis pathway. In contrast, vacuolar membrane‐localised Nyv1 co‐localises with the vacuolar marker Vph1 in EVs (Huh et al. 2003), suggesting its sorting into EVs via vacuole fusion with MVBs or the plasma membrane. Previous studies have demonstrated that Sso2 influences membrane curvature and maturation dynamics, and Nyv1 promotes fusion events that facilitate cargo sorting and release (Furukawa and Mima 2014; Song and Wickner 2017; Starai et al. 2008). Collectively, the findings highlight the significant potential of these proteins as candidate markers for YDEV. (2) Broad conservation and universality. The protein should be stably expressed in exosomes derived from different genotype or phenotype species. In this study, the candidate proteins identified were also detected in the Vesiclepedia dataset (https://www.microvesicles.org), which analysed the protein cargo of EV from wild type and knockout yeast strains, as well as under H_2_O_2_ treatment conditions (Zhao et al. 2019; Chitti et al. 2024). According to Vesiclepedia, the type IV membrane protein Sso2 was absent in YDEVs ONLY when the *vps2/did2* knockout. Vps2/Did2 encodes a Class E Vps protein of the ESCRT‐III complex, which is required for sorting and concentrating proteins that enter invaginating vesicles of the MVB (Banjade et al. 2021). Similarly, another type IV membrane protein Nyv1 was absent in YDEVs derived from several gene mutants, including Vps2, Vps23, Vps36, Bro1, Hse1 and Yca1. The first five items are part of the ESCRT complex and are involved in MVB vesicle formation (Hurley et al. 2025; Larios et al. 2020). Yca1, a Ca^2+^‐dependent cysteine protease, regulates apoptosis upon H_2_O_2_ treatment (Wang et al. 2024). Interestingly, the eisosome component Tos7 was present in all 15 strains examined. Moreover, Tos7 possesses structural features that make it a promising scaffold protein. (3) Large extracellular loop in sorting. The multiple transmembrane protein Tos7 shares a similar structural architecture with the canonical marker protein CD63, featuring two prominent topological loops that map to the vacuolar lumen spanning amino acids 29–88 and the cytoplasmic domain spanning amino acids 110 to 135. Local alignment of the amino acid sequences of Tos7 using the Smith‐Waterman algorithm revealed a 37.4% similarity, which indicates it as a scaffold of YDEVs (Figure S2E).

Detection of conserved trafficking modules (SNAREs, Rabs, ESCRT subunits) in EV preparations aligns yeast EV biogenesis with core eukaryotic trafficking principles while revealing species‐specific remodelling under engineering stress. Proteomics indicates that YDEVs derive from multiple intracellular compartments, including ER, Golgi, endosomes, vacuoles and plasma membrane, which support a model where both ESCRT‐dependent MVB pathways and ESCRT‐independent plasma membrane shedding contribute to the secreted vesicle population (van Niel et al. 2018). Notably, whereas some previous studies reported that depletion of select ESCRT components from fungal EVs (Oliveira et al. 2010; Zhao et al. 2019). But our data reveal condition‐dependent presence and modulation of ESCRT factors, emphasising the dynamic nature of EV composition across physiological states and manipulations. In addition, proteomic trends among Rabs and ESCRT components further suggest coordinated, stress‐responsive traffic reallocation. Most Rab GTPases showed stepwise decreases with increasing engineering burden (F1 > F2 > F3), consistent with graded remodelling of trafficking routes; concomitantly, Vps4 (the ESCRT‐III disassembly ATPase) increased in engineered strains. We propose an ESCRT stress adaptation wherein elevated Vps4 activity accelerates ESCRT‐III turnover and membrane scission (Larios et al. 2020; Quadri et al. 2024), facilitating higher vesicle output when secretory load and cell wall weakness demand increased extracellular release. Together, the SNARE‐Rab‐ESCRT axis appears to be flexibly reprogrammed to balance secretory throughput, fusion efficiency and membrane scission under perturbation.

To summarise, our study integrated proteomic and functional analyses demonstrates that (1) heterologous proteins can be nonspecifically incorporated into YDEVs, (2) EV membranes originate from multiple organelles, and (3) conserved trafficking modules, including ESCRT, Rab GTPases and SNARE complexes, are dynamically remodelled under engineering stress. These findings position *S. cerevisiae* as both a mechanistic model and an engineering chassis for EV production.

## Author Contributions

**Yueyan Li:** conceptualization, methodology, investigation, writing – original draft. **XiaoRan Ma:** conceptualization, methodology, investigation. **Lichao Zhang:** conceptualization, methodology, investigation. **Ning Cao:** resources, visualization. **Zhibo Li:** resources, visualization. **Ruixin Khoo:** resources, visualization. **Mei Wang:** software, data curation. **Changyan Li:** software, data curation. **Deping Hua:** software, data curation. **Xintian Zheng:** writing – review and editing, project administration. **Jinhai Huang:** writing – review and editing, project administration. **Lilin Zhang:** writing – review and editing, project administration. All authors have critically revised the manuscript and approved its publication.

## Funding

This work was supported by the Tianjin Key Science and Technology Support Project under grant 24YFZCSN00180 and Fujian Provincial Science and Technology Program Projects under grant 2025N5009.

## Ethics Statement

The animal study was reviewed and approved by the Institutional Animal Ethical Committee of Institute of Radiation Medicine, Chinese Academy of Medical Sciences and Peking Union Medical College [certificate number: SYXK (Jin) 2024‐0004, Tianjin, China].

## Conflicts of Interest

The authors declare no conflicts of interest.

## Supporting information


**Figure S1:** (A) Heatmap showing abundance changes of processing body (P‐body)‐associated proteins and stress granule assembly related proteins detected in EVs. (B) Heatmap showing abundance changes of ubiquitin‐dependent protein degradation‐related proteins identified in EVs. (C) Subcellular localisation distribution of the top 50 most abundant transmembrane proteins identified in F1‐, F2‐ and F3‐EVs. (D) Venn diagram illustrating the overlap of the top 50 most abundant transmembrane proteins among F1‐, F2‐ and F3‐EVs.

**Figure S2:** (A) Fluorescence microscopy images of wild‐type and recombinant yeast strains expressing N‐ or C‐terminal GFP fusion Sso2 proteins. Scale bar, 10 μm. (B) TEM images showing the morphology of isolated UC‐EVs (scale bar: 500 nm). (C) Detection of target proteins in EVs derived from wild‐type *S. cerevisiae* (BY4741) using tag antibodies against the target proteins. (D) Bottom‐up density gradient separation of UC‐EVs. Western blot analysis of 25 fractions (vol: vol matched) of YDEVs with commercially available anti‐Flag (Cat. No. M20008 Abmart), anti‐HA (Cat. No. M20003 Abmart) and anti‐GFP (Cat. No. T0006 Affinity Biosciences) tags antibodies against indicated proteins. (E) Structural comparison of Tos7 (Q08157, light orange) and CD63 (P08962, blue) using the RCSB PDB comparison tool (https://www.rcsb.org/alignment).

**Table S1:** Yeast strains used in this study.

**Table S2:** Plasmids used in this study.

**Table S3:** Primer list in this study.

**Table S4:** All raw data for heatmap analysis, with different sheet names indicating corresponding image IDs.

## Data Availability

The data that support the findings of this study are available on request from the corresponding author. The data are not publicly available due to privacy restrictions.
