# Supplementary figures and images for: Engineering Yeast Extracellular Vesicle Biogenesis Through Rewiring Membrane Trafficking Pathways
Source: Microb Biotechnol. 2026 Mar 27;19(4):e70338. doi: 10.1111/1751-7915.70338 (PMC13140754; doi:10.1111/1751-7915.70338)

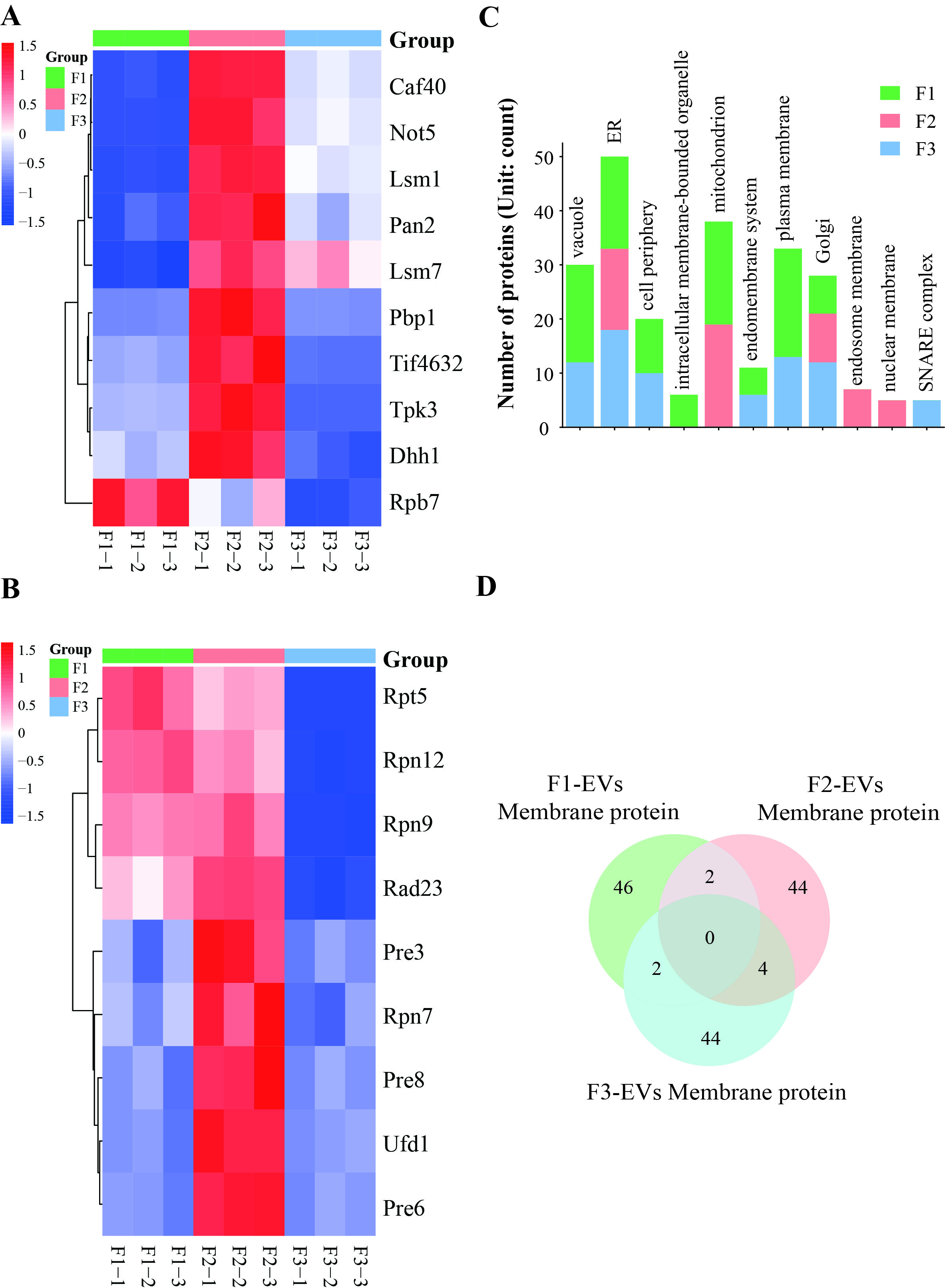

Supplement: Supplementary file 1 — Figure S1: (A) Heatmap showing abundance changes of processing body (P‐body)‐associated proteins and stress granule assembly related proteins detected in EVs. (B) Heatmap showing abundance changes of ubiquitin‐dependent protein degradation‐related proteins identified in EVs. (C) Subcellular localisation distribution of the top 50 most abundant transmembrane proteins identified in F1‐, F2‐ and F3‐EVs. (D) Venn diagram illustrating the overlap of the top 50 most abundant transmembrane proteins among F1‐, F2‐ and F3‐EVs. [file MBT2-19-e70338-s006.jpg]

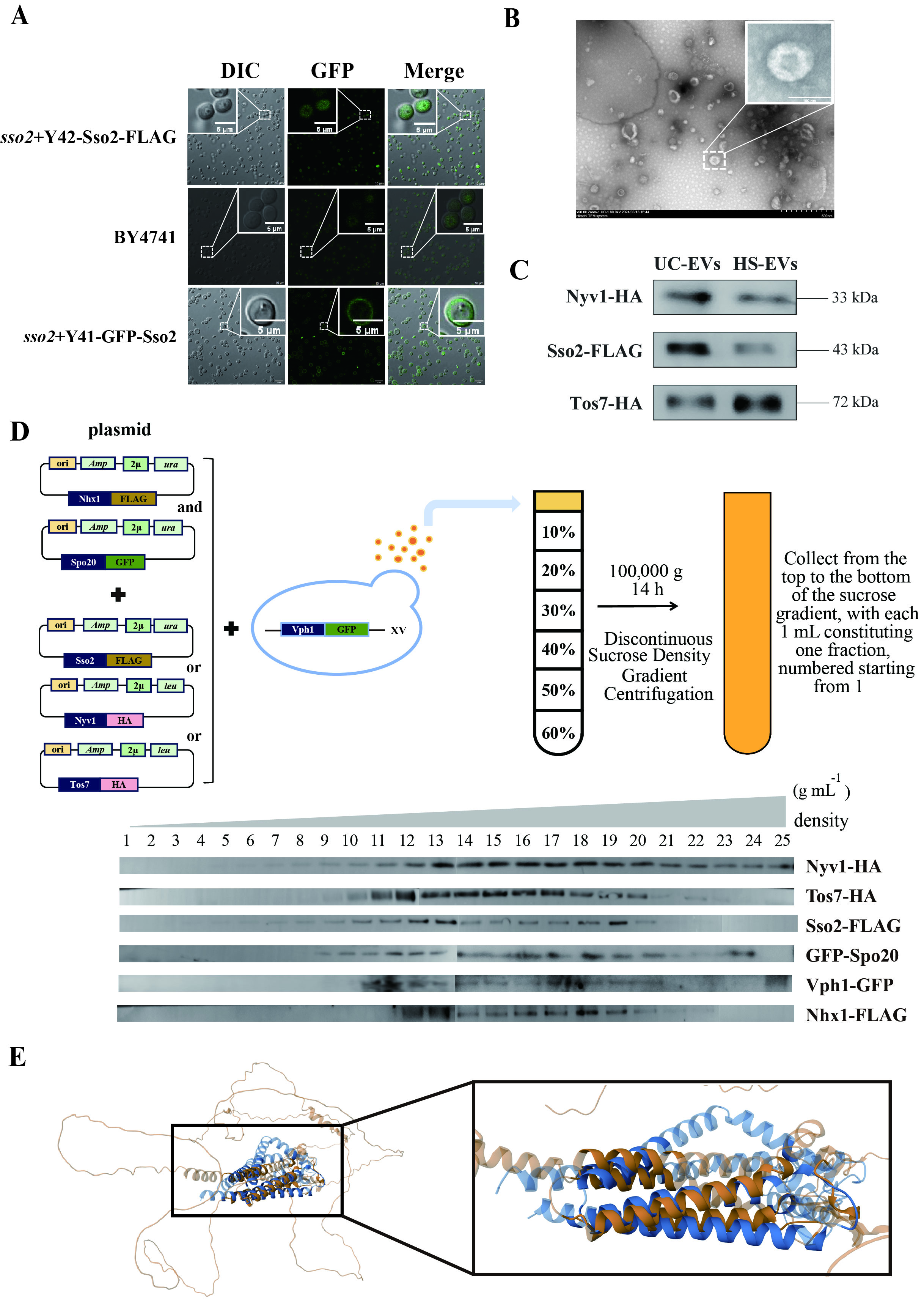

Supplement: Supplementary file 2 — Figure S2: (A) Fluorescence microscopy images of wild‐type and recombinant yeast strains expressing N‐ or C‐terminal GFP fusion Sso2 proteins. Scale bar, 10 μm. (B) TEM images showing the morphology of isolated UC‐EVs (scale bar: 500 nm). (C) Detection of target proteins in EVs derived from wild‐type S. cerevisiae (BY4741) using tag antibodies against the target proteins. (D) Bottom‐up density gradient separation of UC‐EVs. Western blot analysis of 25 fractions (vol: vol matched) of YDEVs with commercially available anti‐Flag (Cat. No. M20008 Abmart), anti‐HA (Cat. No. M20003 Abmart) and anti‐GFP (Cat. No. T0006 Affinity Biosciences) tags antibodies against indicated proteins. (E) Structural comparison of Tos7 (Q08157, light orange) and CD63 (P08962, blue) using the RCSB PDB comparison tool (https://www.rcsb.org/alignment). [file MBT2-19-e70338-s001.jpg]
