# Supplementary material for: Engineering Yeast Extracellular Vesicle Biogenesis Through Rewiring Membrane Trafficking Pathways
Source: Microb Biotechnol. 2026 Mar 27;19(4):e70338. doi: 10.1111/1751-7915.70338 (PMC13140754; doi:10.1111/1751-7915.70338)
Supplement: Supplementary file 3 — Table S1: Yeast strains used in this study. [file MBT2-19-e70338-s005.doc]

**Supplementary Table S1: Yeast strains used in this study**

| Strain name | Genotype | Source |
| --- | --- | --- |
| JDY52-URR1-His-URR2 | *MATa his3Δ200 leu2Δ0 lys2Δ0 trp1Δ63 ura3Δ0 met15Δ0 ho::* URR1-His-URR2 | PMID: 25956650 |
| BY4741 | *MATa his3Δ1 leu2Δ0 met15Δ0 ura3Δ0* | PMID: 29982432 |
| BY4741-Vph1*-*GFP | *MATa his3Δ1 leu2Δ0 met15Δ0 ura3Δ0* Vph1-GFP::HIS3MX6 | PMID: 37888259 |
| F1 / SJDY3 | *MATa his3Δ200 leu2Δ0 lys2Δ0 trp1Δ63 ura3Δ0 met15Δ0 ho::* URR1-His-URR2  ChrXV 160595-161578::sgRNA  ChrXII 234082-235036::sgRNA  ChrIII 136874-137745::sgRNA | This study |
| F2 / SJDY4 | ChrXV 160595-161578::ChiIFN-λ  ChrXII 234082-235036::ChiIFN-λ  ChrIII 136874-137745::ChiIFN-λ  ChrXVI 451173-451645::ChiIFN-λ, TRP | This study |
| F3 / SJDY5 | ChrXV 160595-161578::ChiIFN-λ  ChrXII 234082-235036::ChiIFN-λ  ChrIII 136874-137745::ChiIFN-λ  ChrXVI 451173-451645::ChiIFN-λ, TRP  *chs3::*kanMX4 | This study |
| *tos7Δ* | *tos7::*kanMX4 | Invitrogen Inc. |
| *sso2Δ* | *sso2::*kanMX4 | Invitrogen Inc. |
| *nyv1Δ* | *nyv1::*kanMX4 | Invitrogen Inc. |
