# Supplementary material for: Engineering Yeast Extracellular Vesicle Biogenesis Through Rewiring Membrane Trafficking Pathways
Source: Microb Biotechnol. 2026 Mar 27;19(4):e70338. doi: 10.1111/1751-7915.70338 (PMC13140754; doi:10.1111/1751-7915.70338)
Supplement: Supplementary file 4 — Table S2: Plasmids used in this study. [file MBT2-19-e70338-s002.doc]

**Supplementary Table S2: Plasmids used in this study**

| Plasmid | ORF | Source |
| --- | --- | --- |
| pCut-syn.gRNA | TEF1p-Cas9-CYC1t, SNR52p-syn.gRNA-SUP4t | This study |
| POT1-RFP | RFP | PMID: 25956650 |
| POT-ChiIFN-λ | GPDp-αFactor SP-ChiIFN-λ 6*His-ADH1t | This study |
| Y42 | GFP tag, URA3, 2 micron | Lab stock |
| Y42-FLAG | Flag tag, URA3, 2 micron | This study |
| Y42-Sso2-GFP | SSO2p-Sso2-GFP-CYC1t | This study |
| Y42-Tos7-GFP | TOS7p-Tos7-GFP-CYC1t | This study |
| Y42-Nyv1-GFP | NYV1p-Nyv1-GFP-CYC1t | This study |
| Y42-Sso2-FLAG | SSO2p-Sso2-FLAG-CYC1t | This study |
| Y42-Nhx1-FLAG  Y42-Spo20-GFP | NHX1p-Nhx1-FLAG-CYC1t  Spo20p-Spo20-HA-GFP | This study  Lab stock |
| Y41 | GFP, URA3, CEN | This study |
| Y41-GFP-*Xho*I | GFP, URA3, CEN | This study |
| Y41-GFP-Sso2-ORF | GFP-Sso2-CYC1t | This study |
| Y41-GFP-Sso2 | Sso2p-GFP-Sso2-CYC1t | This study |
| pHAC181 | HA tag, Leu2, 2 micron | Lab stock |
| pHAC181-Tos7-HA | TOS7p-Tos7-HA-CYC1t | This study |
| pHAC181-Nyv1-HA | NYV1p-Nyv1-HA-CYC1t | This study |
| pET28a | His-tag, KanR | Lab stock |
| pET28a-Sso2 | T7p-Sso2-6*His-T7ter | This study |
| pET28a-Tos7 | T7p-Tos7-6*His-T7ter | This study |
| pET28a-Nyv1 | T7p-Nyv1-6*His-T7ter | This study |
