# Supplementary material for: Engineering Yeast Extracellular Vesicle Biogenesis Through Rewiring Membrane Trafficking Pathways
Source: Microb Biotechnol. 2026 Mar 27;19(4):e70338. doi: 10.1111/1751-7915.70338 (PMC13140754; doi:10.1111/1751-7915.70338)
Supplement: Supplementary file 5 — Table S3: Primer list in this study. [file MBT2-19-e70338-s004.doc]

**Supplementary Table S3: Primer list in this study**

|  | **Forward primer (5`-3`)** | **Reverse primer (5`-3`)** |
| --- | --- | --- |
| Y42-Sso2-GFP | gtaaaacgacggccagtgcTACGAGCACACCTTCACA | tcacctttagacattttgcaCTTTCTTGTTTCCACAACGG |
| Y42-Tos7-GFP | gtaaaacgacggccagtGTGTACGCAATTTCTCTTATCCT | tcacctttagacattttgcaCCTAAATCCGTAAGGATTGTTC |
| Y42-Nyv1-GFP | cgacctgcaggcatgCAACGACAAGCAATACGGA | cacctttagacattttgcatgcCCACAGATAGAAAAACAT |
| Y42-FLAG-dele GFP | ctagagactacaaagacgatgacgacaagTAAGGTACGTACCAAGATGGCC | GTACCTTACTTGTCGTCATCGTCTTTGTAGTCTctagaggatccccgggt |
| Y42-Sso2-FLAG | gtaaaacgacggccagtgCTACGAGCACACCTTCACA | cgtcatcgtctttgtagtcCTTTCTTGTTTCCACAACGGA |
| Y42-Nhx1-FLAG | gtaaaacgacggccagtGGTTGCTATTAGCATCGACGCCG | tcgtcatcgtctttgtagtcGTGGTTTTGGGAAGAGAAATC |
| anti-F/R | gatgaattgtacaaaCTCGAGggtacgtaccaagatg | TTTGTACAATTCATCcatacc |
| GFP-Sso2-Fo / Ro | gatgaattgtacaaaCTCGAGATGAGCAACGCTAATCCT | gggcctgtttactcaCTCGAGCTTTCTTGTTTCCACAAC |
| GFP-Sso2-Fp / Rp | aaaacgacggccagtGAATTCGCTACGAGCACACCTTCA | acctttagacatTTTGAATTCTGCTGCAATATTTGTGCG |
| PHAC181-Tos7-HA | gtaaaacgacggccagtgTGTACGCAATTTCTCTTATCCT | caggaacatcgtatgggtaCCTAAATCCGTAAGGATTGTTCA |
| PHAC181-Nyv1-HA | ttgtaaaacgacggccagtgAACGACAAGCAATACGGAACC | caggaacatcgtatgggtaCCACAGATAGAAAAACATGAAAGC |
| Nyv1-dele intron | TGAAACGCTTTAATGTAAGTTATGTGGAAGTTATA | CATTAAAGCGTTTCATTT |
| pET28a | CTCGAGCACCACCACCACC | GAATTCGGATCCGCGACC |
| pET28a-Sso2 | tgggtcgcggatccgaattcATGAGCAACGCTAATCCTTATGAG | tggtggtggtggtgctcgagTTACTTTCTTGTTTCCACAACGGA |
| pET28a-Tos7 | tgggtcgcggatccgaattcGAAAAGGGCATGCTGCTGAA | tggtggtggtggtctcgagTTACCTAAATCCGTAAGGATTGTTCA |
| pET28a-Nyv1 | tgggtcgcggatccgaattcATGGTCTTAGTAAGTTATGTGGAAGTTATAAA | tggtggtggtggtgctcgagTTACCACAGATAGAAAAACATGAAAGC |
| pCut | GGCTAGTCCGTTATCAACTTG | GATCATTTATCTTTCACTGCG |
| ChrXV 160595-161578::sgRNA | TAAAGCGTCGCGCAATCGAGGAGGGGGTAACACCCCTCCGCGCTCT | CCTCCTCGATTGCGCGACGCTTTACTTTAATTAATATATGT |
| ChrXII 234082-235036::sgRNA | AAAGCGTCGCGCAATCGAGGAGGTAAACTTCAACACCTTATAT | CCTCCTCGATTGCGCGACGCTTTGGGTGTGTTTATTACATG |
| ChrIII 136874-137745::sgRNA | CCTCCTCGATTGCGCGACGCTTTGAAAAAGGAAGTGTTT | AAAGCGTCGCGCAATCGAGGAGGGGGTGAAGCCAAAATAAAGG |
| ChrXV 160595-161578::ChiIFN-λ | CACTTCTCCTATGCACATCATTATCAATACTGCCATT | CGCGGAGGGGTGTTACCCCCGGTAGAGGTGTGGT |
| ChrXII 234082-235036::ChiIFN-λ | CATGTAATAAACACACCCTCATTATCAATACTGCCAT | AAGGTGTTGAAGTTTACCGGTAGAGGTGTGGTC |
| ChrIII 136874-137745::ChiIFN-λ | CTTTATTTTGGCTTCACCCTCATTATCAATACTGCCAT | AGGGAAACACTTCCTTTTTCCCGGTAGAGGTGTGGTCAAT |

*Lowercase letter represent homologous arm sequences.
